# In Silico Analysis of s-DAPK-1: From Structure to Function and Regulation

**DOI:** 10.3390/cimb47060416

**Published:** 2025-06-04

**Authors:** Lilian Makgoo, Salerwe Mosebi, Zukile Mbita

**Affiliations:** 1 Department of Biochemistry, Microbiology and Biotechnology, University of Limpopo, Private Bag X1106, Sovenga 0727, South Africa; makgoolilian@gmail.com; 2 Department of Life and Consumer Sciences, University of South Africa, Private Bag X06, Florida 1710, South Africa; mosebs@unisa.ac.za

**Keywords:** s-DAPK-1, in silico, microRNAs, hydropathy, 3D structure, thermodynamics, gene regulation

## Abstract

The existence of s-DAPK-1, an alternatively spliced variant of DAPK-1, adds complexity to our understanding of the proteins involved in the regulation of cell survival, apoptosis, and autophagy. DAPK-1 has been implicated in the regulation of these processes; however, it remains unclear whether s-DAPK-1 also plays a similar role or a separate function; thus, determining its involvement in these processes is challenging due to the limited understanding of its regulation, interacting partners, function, and three-dimensional (3D) structure. Hence, this study was aimed at (1) understanding the regulation of s-DAPK-1 by predicting its microRNA targets, (2) predicting the 3D structure of s-DAPK-1, (3) its physicochemical and thermodynamic properties, (4) its interacting partners, and (5) molecular functions using computational methods. To achieve this aim, various bioinformatics tools and in silico webservers, such as ProteinPrompt, ProtParam, ProtScale, ScooP, Hawkdock, Phyre2, I-TASSER, PSIPRED, SAVES, and PROCHECK, along with user-friendly databases, such as NCBI, TarBase, and Protein Data Bank (PDB), were employed. For miRNA prediction, we used TarBase, and identified the specific microRNAs targeting s-DAPK-1. Furthermore, the Phyre2 database demonstrated that s-DAPK-1 possesses 40% alpha helices and 4% beta strands, forming a stable 3D structure. Additionally, s-DAPK-1 demonstrated stability to withstand high temperatures, suggesting that it is a thermostable protein. Moreover, s-DAPK-1 was found to interact with a variety of proteins involved in tumor progression and gene regulation, including a prion protein and histone H2B type 2-E (H2B2E). This suggests that s-DAPK-1 may perform diverse molecular functions such as regulation of metabolic processes, nucleic acid binding, and mRNA splicing by interacting with different proteins.

## 1. Introduction

Progressively, substantial bioinformatics databases continue to provide new knowledge on biology, and it has emerged as a discipline to interpret experimental data in order to better understand biological processes using the information contained within databases [1,2]. Creating and answering biological questions that were previously considered too complex has been made possible by the enormous amount of data contained within these databases. Specifically, in the field of proteomics, bioinformatics plays an important role in all aspects of protein analysis, including sequence analysis, structure analysis, and evolution analysis [3,4]. For example, bioinformatics tools predicted death-associated protein kinase 1 (DAPK-1) as the most promising Alzheimer’s disease (AD)-related target for quercetin [5]. Moreover, bioinformatics tools have shed light on potential DAPK-1 inhibitors [6,7], DAPK-1’s functional role [6], and the proteins it interacts with [8]. This demonstrates the ability of bioinformatics tools to resolve questions in the field of proteomics. However, despite the availability of datasets retrieved from bioinformatics tools for various less-studied proteins, including s-DAPK-1, a comprehensive summary of these datasets remains lacking.

DAPK-1, a protein kinase with multiple domains, plays roles in various cellular processes, including autophagy, apoptosis, and cell survival signaling pathways [9]. Additionally, the *DAPK-1* gene undergoes alternative splicing to produce a short isoform referred to as s-DAPK-1 [10]. The regulation of DAPK-1 protein levels is intricately managed through a combination of transcriptional and post-transcriptional processes. Notably, transcription factors such as p53 and cAMP response element-binding protein 1 (CREB1) play significant roles in modulating the transcription of *DAPK-1* [11]. DAPK-1 also has many other downstream targets, including N-methyl-D-aspartate receptor 2B (NR2B) [12], peptidyl–prolyl cis–trans isomerase NIMA-interacting 1 (Pin1) [13], and DEAD-box helicase 20 (DDX20) [14]. Targeting these downstream targets of DAPK-1 can alleviate stroke damage, obstruct the ability of Pin1 to activate oncogenic transcription factors, and suppress cell proliferation and tumor development [12,13,14].

Moreover, *DAPK-1* has been shown to be post-transcriptionally regulated by a group of noncoding RNA molecules known as microRNAs (miRs) [15,16,17]. These miRs bind to their target mRNAs through sequence complementarity, leading to cleavage of mRNA and translation repression [18]. Several microRNAs have been identified as regulators of DAPK1, including miR-143-3p in AD [19], miR-191 in endometriosis-associated ovarian cancer [20], and miR-483-5p in nasopharyngeal carcinoma [21]. In addition, bioinformatics tools have also been applied to predict microRNAs targeting *DAPK-1*, such as miR-141-3p in polycystic ovary syndrome [22]. However, there is currently no research study that has examined how microRNAs regulate s-DAPK-1, a variant produced by the alternative splicing of DAPK-1.

Alternative splicing (AS) is a key process in generating proteome diversity, with projections suggesting that 95% of genes are alternatively spliced in humans [23,24,25]. Alternative splicing allows a single gene to generate multiple unique mRNA transcripts, which can subsequently be translated into different protein isoforms. These isoforms may vary in their structures, activities, and interactions, contributing to a variety of functional roles within a cell or tissue [26,27]. According to the Ensembl website, there are 20 splice variants of *DAPK-1* gene, with 7 of them potentially coding for proteins and 13 lacking coding capacity [28]. To date, laboratory experiments have validated the existence of only the s-DAPK-1 spliced variant [10]. An examination of multiple sequence alignments indicates that approximately 22% of the amino acids in s-DAPK-1 are identical to those making up the DAPK-1 isoform.

**Figure 1:**
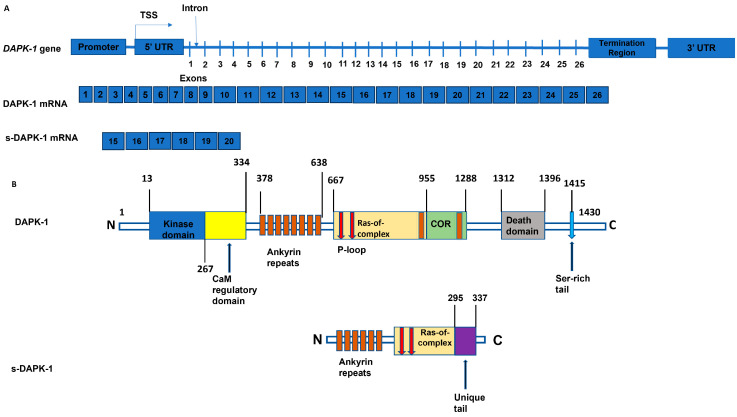
Illustration of the *DAPK-1* gene. (**A**) shows transcribed mRNAs consisting of exons numbered 1 to 26 for the wildtype DAPK-1 transcript and 15 to 20 exons for the s-DAPK-1 transcript. (**B**) shows various domains and motifs found in wildtype DAPK-1 (amino acids 1 to 1430) and the s-DAPK-1 isoform, which has 337 amino acids, yielding ankyrin repeats, Ras-of complex domain and a unique C-terminal tail, lacking the Serine-rich tail, which is indicated by a blue arrow.

Expression of the s-DAPK-1 mRNA starts on introns 13–14 within the *DAPK-1* gene and ends on introns 20–21 within the *DAPK-1* gene (Figure 1). The start codon, AUG, for this mRNA is located between the 10–12 bases of exon 15 within the *DAPK-1* gene, making its translation in-frame with DAPK-1 mRNA [10]. Further, s-DAPK-1 shares exons 15, 16, 17, 18, 19, and 20 with the full-length DAPK-1 mRNA, and its stop codon is located within base pairs 124–126 of intron 20–21; s-DAPK-1 mRNA encodes a 337 amino acid polypeptide encompassing part of the ankyrin-repeat domain, the P-loop motifs, part of the cytoskeletal binding domain of DAPK-1, and a unique C-terminal “tail” extension of 42 amino acids that is absent in DAPK-1 (Figure 1). Based on the Blastp results, this 42-amino acid query sequence was only mapped on s-DAPK-1 but not to any other motif or domain found in the *Homo sapiens* database. Interestingly, Lin et al. [10] showed that this s-DAPK-1 C-terminal tail features an internal proteolytic processing site, and the deletion of this site increased the s-DAPK-1 stability and augmented the membrane-blebbing effect of s-DAPK-1. However, there is still limited literature on the structure, protein–protein interactions, cellular function, and expression of s-DAPK-1 in health and disease.

Lin et al. [10] documented that when comparing the expression of s-DAPK-1 mRNA in cancerous cells (HCT116 and A375) and non-cancerous cells (HEK-293), s-DAPK-1 was highly expressed in HEK-293 (5.517-fold change) relative to HCT116 (0.000-fold change) and A375 (0.455-fold change) cells, indicating that s-DAPK-1 could potentially serve as an antitumor protein, particularly in the cases of skin and colorectal cancer. Given that s-DAPK-1 is an isoform of DAPK-1, it has the potential to contribute to various biological processes. Therefore, there is a significant interest in understanding its 3D structure, function, and regulation. The 3D structure, function, and regulation of DAPK-1 are extensively documented in both physiological and pathological conditions. However, knowledge regarding the 3D structure, function, and regulation of s-DAPK-1, an isoform of DAPK-1, remains unexplored. Even studies on the application of bioinformatics to predict s-DAPK-1’s 3D structure, function, and regulatory mechanism are yet to be conducted. The question of whether s-DAPK-1 mimics DAPK-1’s 3D structure, function, and regulation remains unanswered. The application of bioinformatics holds the potential to address all the critical questions regarding s-DAPK-1.

## 2. Materials and Methods

### 2.1. Prediction of microRNAs (miRs) Targeting s-DAPK-1 mRNA

The TarBase (https://dianalab.e-ce.uth.gr/tarbasev9, accessed on the 24 April 2024) database was used to search for microRNAs targeting s-DAPK-1 [29]. The Tarbase database was searched using “DAPK1” under the “interactions” option. The results were filtered to display only high-confidence miRNAs (validated through experimental data), with a microT prediction score of 0.8 and higher and expressed within *Homo sapiens*. Further confirmation of these miRNAs as targets of DAPK1 was through the use of miRWalk (http://mirwalk.umm.uni-heidelberg.de/, accessed on 24 April 2024) and TargetScan (https://www.targetscan.org/vert_80/, accessed on 24 April 2024). These microRNAs were mapped onto the s-DAPK-1 mRNA sequence to identify miRNAs targeting s-DAPK-1.

### 2.2. Protein Sequence Retrieval

The s-DAPK-1 protein sequence was retrieved from the NCBI database (https://www.ncbi.nlm.nih.gov/, accessed on the 9 March 2024), which is a freely accessible database containing data on protein sequences and their functional annotation [30]. The s-DAPK-1 protein sequence was retrieved using its accession number AK127855.1 in the FASTA format. The protein sequence was then used to predict s-DAPK-1’s hydropathy plot, physicochemical parameters, interacting partners, molecular functions, secondary and tertiary structures.

### 2.3. Modeling and Validation of s-DAPK-1’s 3D Structure

The s-DAPK-1 protein sequence in the FASTA format was then deposited in the Phyre2 database (http://www.sbg.bio.ic.ac.uk, accessed on 15 April 2024) [31] to generate the secondary structure of s-DAPK-1 using the normal modeling mode. The 3D structure of s-DAPK-1 was modeled using the I-TASSER On-line Server (https://zhanggroup.org/I-TASSER/, accessed on 15 April 2024) [32,33]. The quality of the resultant model was assessed using the C-score and the TM-score, and its validation was conducted through the Ramachandran plot on PROCHECK under the SAVES server (https://saves.mbi.ucla.edu/, accessed on 19 April 2024) [34].

### 2.4. Prediction of Physical and Chemical Parameters of s-DAPK-1

The ProtParam tool (https://web.expasy.org/protparam/, accessed on 11 May 2024) [35] was used to estimate physicochemical parameters of s-DAPK-1. The raw protein sequence of s-DAPK-1 was used to query the server. The server provides directly calculated values of pI (isoelectric point), molecular weight, percentage of each amino acid, extinction coefficient, instability index, aliphatic index, and grand average of hydropathicity (GRAVY).

### 2.5. Prediction of s-DAPK-1’s Hydrophobicity and Thermodynamic Parameters

The hydropathy plot of s-DAPK-1 was generated using the default settings of ProtScale (https://web.expasy.org/protscale/, accessed on 17 May 2024) [36]. This tool allowed for a quantitative analysis of either the hydrophobic or hydrophilic nature of the amino acids present in the protein sequence of s-DAPK-1. Furthermore, the ScooP tool (http://babylone.3bio.ulb.ac.be/SCooP/k_query.php, accessed on 17 May 2024) [37] was used to predict the thermodynamic characteristics of s-DAPK-1 under varying temperature conditions. To query the ScooP tool, the predicted 3D structure of s-DAPK-1 was used.

### 2.6. Prediction of Protein-Protein Interactions Involving s-DAPK-1

The ProteinPrompt (https://proteinformatics.uni-leipzig.de/protein_prompt/, accessed on 10 April 2024) online sequence-based approach was used to predict proteins interacting with s-DAPK-1. This webserver uses binding scores to indicate high confidence in binding. The quality and speed of this system make it a suitable high-throughput method for scanning sequence libraries [38].

### 2.7. Protein–Protein Docking

The ProteinPrompt results were confirmed through the use of HawkDock (http://cadd.zju.edu.cn/hawkdock/, accessed on 16 April 2024), a template-free blind protein–protein docking tool. HawkDock was used in order to predict the binding affinity between the proteins identified by ProteinPrompt and the s-DAPK-1 protein. HawkDock is a useful tool for predicting the structures of protein complexes that are yet to be experimentally determined [39]. In order to carry out the HawkDock analysis, the ProteinPrompt results were refined to include solely proteins with confirmed 3D structures stored in the Protein Data Bank (https://www.rcsb.org/, accessed on 27 April 2024). These proteins were cleaned using the Discovery Studio visualizer. The optimal pose was determined by considering the free binding energy, where a more negative score indicated a higher binding energy strength. The ideal conformation was submitted to the Protein Interaction Analysis tool of BioLuminate to visualize the amino acids and bonds participating in the interactions between the two proteins.

### 2.8. Prediction of the Functions of the Modeled s-DAPK-1 Structure

The PSIPRED (http://bioinf.cs.ucl.ac.uk/psipred/, accessed on 16 May 2024) webserver has access to FFPred 3, a tool to predict protein function. To make predictions, this webserver uses a probability score and support vector machine (SVM) reliability. SVMs within FFPred 3 were used to scan the s-DAPK-1 input sequences in order to make function predictions. Each SVM examines the relationship between protein function and biophysical properties [40]. The results were refined to display the first 25 molecular functions and biological processes related to s-DAPK-1, ranked by reliability and probability score. The confidence in assigning the function to the protein is determined by the posterior probability. A higher-reliability SVM model, denoted by “H”, is associated with greater confidence. In the default view of the FFPred results tab, the predictions of functions from higher-reliability SVM models (“H”) are prioritized and listed first. A lower-reliability SVM model, denoted by “L”, is associated with a low confidence score; the predictions of functions from lower-reliability SVM models (‘L’) are listed last under the FFPred results tab.

## 3. Results

### 3.1. Proteins s-DAPK-1 and DAPK-1 Are Regulated by miRs

The expression and regulation of DAPK-1 are well-documented in different diseases, including cancer; however, there is a notable lack of information regarding the expression and regulation of s-DAPK-1 in various diseases, particularly in cancer. Consequently, it is crucial to investigate the molecular mechanism that could potentially regulate the expression of s-DAPK-1. Therefore, the TarBase tool was used to predict microRNAs that can regulate s-DAPK-1. Currently, there are no available data on s-DAPK-1 regulation by microRNAs; thus, “DAPK-1” was used to search various webservers. Only high-confidence miRNAs with a microT prediction score of 0.8 and higher were considered.

In the analysis of TarBase data (Supplementary Figure S1), the microT prediction score serves as an indicator of the accuracy of the prediction, with a score close to 1 indicating a higher level of confidence. A total of 4 miRs featured in Supplementary Figure S1 were identified to target DAPK-1. The mapping of the microRNA binding site was conducted on the mRNA sequence of s-DAPK-1. Interestingly, the analysis revealed that certain DAPK-1 microRNAs may also target s-DAPK-1 (Table 1). Additionally, it can be inferred from these findings that microRNAs exhibit binding not only to the 3′ UTR of the mRNA, as the identified microRNAs only formed complementary bases with the nucleotides present in the 5′ end of s-DAPK-1 (Table 1). The sequences of these miRs, together with their target sequence on s-DAPK-1 and DAPK-1, are illustrated in Supplementary Figure S2.

### 3.2. The 3D Structure of s-DAPK-1 Contains Ankyrin Repeats

**Figure 2:**
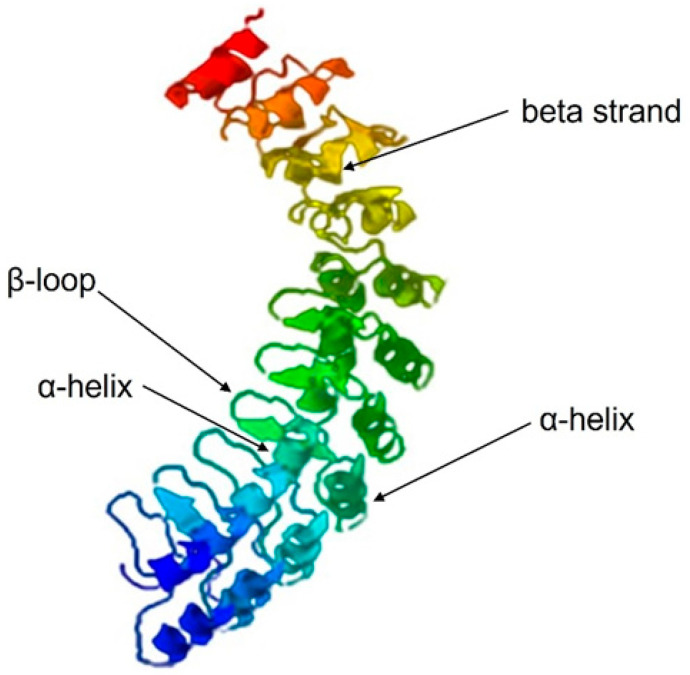
Modeled 3D structure of s-DAPK-1 predicted using the I-TASSER tool. The structure of the model exhibits few beta strands and antiparallel α-helices, which are separated by loops and arranged in a nearly parallel manner. On the predicted protein structure, the red secondary structures are at the c-terminus of s-DAPK-1 while the blue structures are at N-terminus. The structure is represented in a ribbon model.

Once the structure of a protein is comprehended, it is easy to understand its function and its interaction partners, thus gaining insights into various biological interactions and cellular functions. Therefore, the Phyre2 webserver was used to predict the s-DAPK-1 secondary structure (Supplementary Figure S3), followed by the prediction of its tertiary structure using I-TASSER. The I-TASSER webserver is ranked as the top method in the community-wide Critical Assessment of Protein Structure Prediction (CASP) experiments for accurate 3D structure prediction [16]. I-TASSER modeled five (5) s-DAPK-1 structures (Supplementary Figure S4), which differ in their C-scores (0.54, −2.17, −1.20, −2.03, −3.96). The C-score quantitatively measures the confidence of each model, and it is determined by calculating the significance of threading template alignments and the convergence parameters of structure assembly simulations. The C-score should fall within the range from −5 to 2, where a higher C-score indicates a model with greater confidence, while a lower C-score suggests the opposite. Therefore, model 1 (Figure 2) qualified to be the best-modeled 3D structure of s-DAPK-1 due to its highest confidence score of 0.54. Based on Figure 2, s-DAPK-1 does not seem to resemble characteristics of a globular protein, which are generally spherical in shape, resulting from the folding of a polypeptide chain into a compact structure. In contrast, s-DAPK-1 is made of two antiparallel α-helices and a β-loop, which are arranged in a manner that creates an L-shaped domain that resembles a cupped hand. Such structural characteristics are typical of ankyrin repeat-containing proteins, which are considered fibrous proteins.

Validation of the predicted model was carried out using the Ramachandran plot, which identifies amino acid distribution in different secondary structure regions. Based on Supplementary Figure S5, 72.3% of amino acids are found in favorable regions, while 21.8% are in additional allowed regions, 3.1% are in generously allowed regions, and only 2.8% are in disallowed regions. Based on these results, most residues fall within the favored regions.

### 3.3. Physicochemical Characterization of s-DAPK-1

The three-dimensional structure and biological activity of proteins are influenced by the physicochemical characteristics of the amino acids they are composed of. Therefore, it was deemed necessary to assess the physicochemical properties of s-DAPK-1. The physicochemical parameters of s-DAPK-1 were computed using Expasy’s ProtParam tool. As depicted in Table 2 (physicochemical properties of s-DAPK-1), the calculated isoelectric point (8.96 > 7) for s-DAPK-1 demonstrated its basic nature, while the extinction coefficient at 280 nm varied from 27,680 to 26,930 M^−1^ cm^−1^, suggesting a higher concentration of tyrosine (Tyr) and tryptophan (Trp) amino acids. These findings suggest that UV spectral methods can be utilized to quantify the s-DAPK-1 protein because it has aromatic tryptophan and tyrosine residues, which exhibit strong UV light absorption at 280 nm. Additionally, the stability of s-DAPK-1 was also assessed using the instability index value, an estimation of the stability of a protein in a test tube. Stable proteins are predicted to have an instability index below 40; therefore, s-DAPK-1 is considered unstable because its instability index is predicted to be 40.67.

Furthermore, the aliphatic index of s-DAPK-1, representing the proportion of aliphatic side chains (alanine, valine, isoleucine, and leucine), was measured to be 87.74 for s-DAPK-1. An exceptionally high aliphatic index of 87.74 suggests that s-DAPK-1 could remain stable across various temperature ranges. This is because the aliphatic index reflects the relative volume occupied by aliphatic amino acid side chains, and hydrophobic interactions between these residues help to stabilize the protein structure, making it more resistant to unfolding or denaturation at higher temperatures, thus contributing to protein stability. The GRAVY value represents the ratio of hydropathy values of amino acids to the number of residues in the sequence. For s-DAPK-1, the predicted score was −0.216, suggesting its potential classification as a hydrophilic protein.

**Figure 3:**
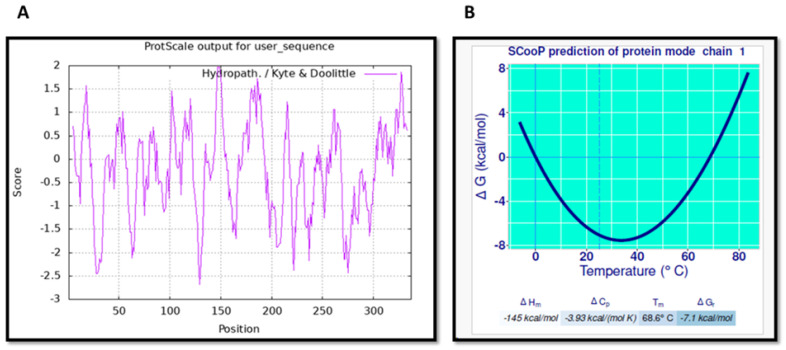
(**A**) The hydropathy plot of s-DAPK-1 displays peaks with a positive score representing hydrophobic regions within the protein sequence, while peaks with a negative score signify hydrophilic regions; (**B**) s-DAPK-1 protein stability curve defined by ΔG as a function of temperature. The solid line on the protein stability curve indicates the transition of s-DAPK-1 from stable to unfolded state.

To detect the patches of hydrophobic amino acid residues in s-DAPK-1, a hydropathy plot was generated using the ProtScale tool. The plot provided insight into the degree of hydrophobicity or hydrophilicity of s-DAPK-1 amino acids. The graph in Figure 3A shows numerous prominent negative peaks (hydrophilic) and a few strong positive peaks (hydrophobic). Additionally, the thermodynamic properties associated with the folding transition of the s-DAPK-1 protein were assessed. The calculated values included a melting temperature (T_m_) of 68.6 °C, a standard folding enthalpy (ΔHm) of −145 kcal/mol, and a standard folding heat capacity (ΔC_p_) of −3.93 kcal/(mol K). All these data suggest that s-DAPK-1 may exhibit thermodynamic stability. The stability profile of the s-DAPK-1 protein, represented by the change in Gibbs free energy (ΔG) in relation to temperature (Figure 3B), demonstrated its potential to remain stable even at higher temperatures, as evidenced by a negative ΔG value.

### 3.4. Protein s-DAPK-1 Interacts with Other Proteins

To further understand the role of s-DAPK-1, the proteins that interact with s-DAPK-1 were investigated using the ProteinPrompt tool, utilizing only the s-DAPK-1 protein sequence to query the server. The first 10 binding partners of s-DAPK-1 with high binding scores were analyzed (Table 3). A significant number of proteins identified in Table 3 are implicated in gene regulation and cancer progression. Notable examples of these proteins include histone H2B type 2-E, prion protein, nuclear protein, polyubiquitin-B, and epididymis secretory protein Li 50. It is possible that s-DAPK-1 interacts with proteins identified in Table 3 proteins using ankyrin repeats, because variations within the surface exposed residues in ankyrin repeat motifs/domains enable specific protein binding.

**Figure 4:**
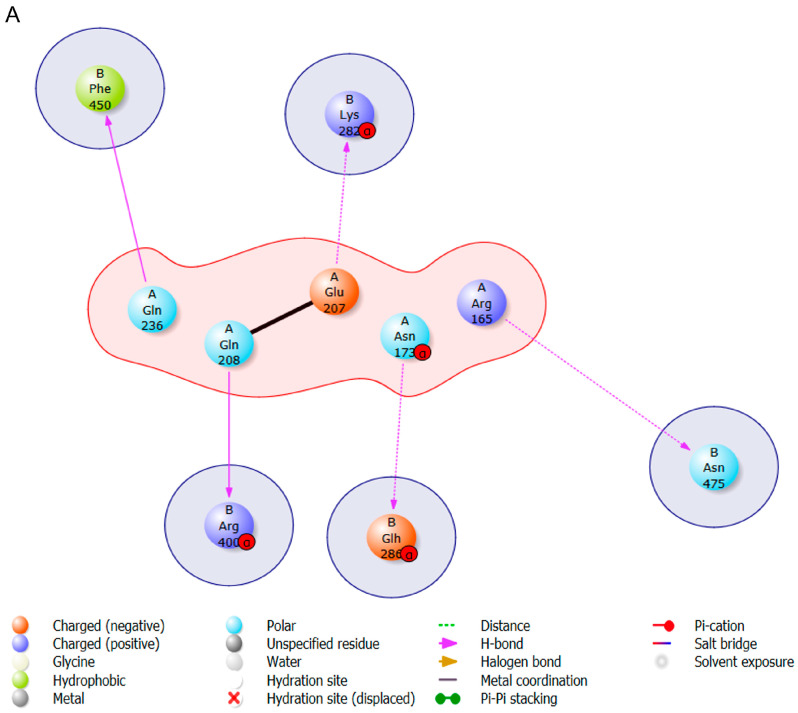

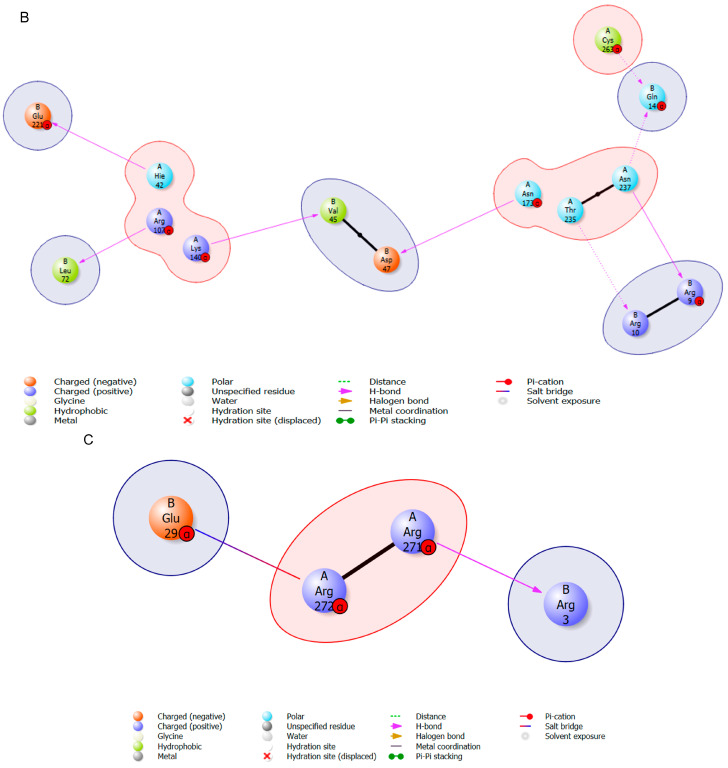

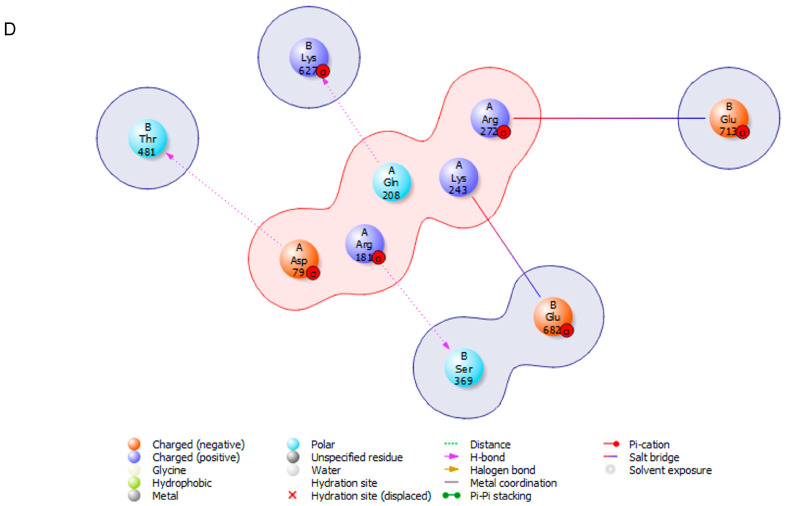
Binding energies and visualization of amino acids involved in protein–protein interactions. The s-DAPK-1 and polyubiquitin-B pose (**A**) has a binding energy of −6837.03 kcal/mol. The s-DAPK-1 and microtubule-associated proteins 1A/1B light chain 3B pose (**B**) has a binding energy of −7625.15 kcal/mol. The s-DAPK-1 and dynein light chain 1 pose (**C**) with a binding energy of −4944.96 kcal/mol. The s-DAPK-1 and histone H2B type 2-E pose (**D**) with a binding energy of −7383.30 kcal/mol.

In order to verify whether the proteins predicted by ProteinPrompt exhibit any binding affinity to s-DAPK-1, blind protein–protein docking (Figure 4) was conducted. Ten binding poses were predicted for each protein docked on s-DAPK-1, and subsequently, the pose with the highest binding energy was chosen from these predictions. It is important to highlight that there is no universally accepted standard value for evaluating the docking score. The score can only be deemed as favorable or unfavorable based on another reference value obtained from the same protein. Some of the proteins identified by ProteinPrompt exhibited a binding affinity to s-DAPK-1 as shown by negative Gibbs free energy. This statement is made because we did not dock all the proteins, but rather focused on those with solved structures and smaller sizes that can function as ligands. The docked proteins were polyubiquitin-B, dynein light chain 1, microtubule-associated proteins 1A/1B light chain 3B and histone H2B type 2-E. The functions of these proteins are already highlighted in Table 3; all these proteins may have the potential to bind to s-DAPK-1. Upon examining the binding energies (Figure 4), it became apparent that microtubule-associated protein 1A/1B light chain 3B exhibited the highest binding affinity, with the binding free energy of −7625.15 kcal/mol. Following this, histone H2B type 2-E demonstrated the next highest affinity, with the binding energy of −7383.30 kcal/mol, succeeded by polyubiquitin-B with −6837.03 kcal/mol. Lastly, dynein light chain 1 is identified as the protein with the lowest binding affinity towards s-DAPK-1 because of its binding energy of −4944.96 kcal/mol.

The next step was to visualize the amino acids involved in protein–protein interactions as well as the bonds implicated. The presence of hydrogen bonds is crucial for the stability of protein–ligand complexes. As ligands interact and form more hydrogen bonds with the protein, the overall stability of the protein–ligand complex is enhanced. Figure 4 provides evidence of specific amino acids that play a role in the binding process through hydrogen bonds. In thes-DAPK-1 and polyubiquitin-B complex, 5 hydrogen bonds were identified between specific residues; namely, Gln208–Arg400, Glu207–Lys282, Asn173–Glu286, Arg165–Asn475, and Gln236–Phe450. The dynein light chain 1 and s-DAPK-1 complex exhibited the formation of one hydrogen bond between Arg271 and Arg3. Furthermore, the microtubule-associated proteins 1A/1B light chain 3B and s-DAPK-1 complex displayed 8 hydrogen bonds; the amino acids involved in these bonds are shown in Figure 4. Lastly, the histone H2B type 2-E and s-DAPK-1 complex formed three hydrogen bonds involving residues Asp79–Thr481, Arg181–Ser369, and Gln208–Lys627. To sum up, the 8 hydrogen bonds found in the microtubule-associated proteins 1A/1B light chain 3B and s-DAPK-1 complex establish it as the most stable complex among others.

### 3.5. Protein s-DAPK-1 Performs Various Cellular Functions

Currently, there is limited information on s-DAPK-1, both structurally and functionally. The protein–protein interactions in this study demonstrated that s-DAPK-1 may interact with numerous proteins (Table 3 and Figure 4). Therefore, it was fitting to further investigate probable s-DAPK-1 functions, and this was achieved utilizing the FFPred server. Upon examining the proteins that interact with s-DAPK-1 and their impact on its function, it was discovered that s-DAPK-1 may form interactions with H2B2E (Table 3), a crucial component of the nucleosome structure. Therefore, it is unsurprising that the predicted functions of s-DAPK-1 include nucleic acid binding, RNA binding, regulation of the RNA biosynthetic process, regulation of the metabolic process, and adenyl nucleotide binding, as presented in Table 4a. Based on the information that is shown in Table 4a,b, it is evident that s-DAPK-1 and DAPK-1 may have comparable functions such as the regulation of nitrogen compounds’ metabolic processes, catalytic activity, and adenyl nucleotide binding activity. Contrary to s-DAPK-1, DAPK1 plays a crucial role in regulating various activities linked to the central dogma. Among the functions of DAPK-1 presented in Table 4b, its role in the regulation of metabolic processes is particularly noteworthy because it is likely to be through this function that DAPK-1 facilitates different forms of cell death, such as apoptosis and autophagy.

## 4. Discussion

Several complete and draft genomes are now available, and these include the human genome; however, the biggest task and gap is assigning gene product functions, interactions, regulation, and structures [60,61,62]. To analyze the protein sequence, function, structure, motif/domain, regulation, and interaction network, several bioinformatics tools have been developed and made publicly available [29,63,64,65].

Data regarding the regulation of s-DAPK-1 in cancer cells is unavailable, particularly in relation to its posttranscriptional regulation. In contrast to s-DAPK-1, there is considerable evidence in the literature on microRNAs targeting DAPK-1; for example, DAPK-1 has been reported to be modulated by miR-26b-5p and miR-632 microRNAs in the contexts of intestinal ischemia and gastric cancer, respectively [65,66,67]. Interestingly, this study successfully predicted microRNAs targeting s-DAPK-1 (Table 1) using the TarBase tool; these results were obtained following a query performed on the server using the *DAPK-1* gene. “*DAPK-1*” but not “s-DAPK-1” was used to query the microRNA databases because the identification and characterization of miRNA targets rely on computational prediction methods that often use the full gene sequence, rather than specific splice variants.

In addition, s-DAPK-1 starts in intron 13–14 of DAPK-1, thus classifying it as an intron-retained transcript. Generally, intron-retained transcripts have unique 3′ and 5′ UTRs that differ from those found in the main gene’s spliced transcript [68,69]. However, despite changes in the 3′ UTR and 5′ UTR regions of intron-retained transcripts, these transcripts can still feature miRNA binding sites that are also present in the main gene’s transcript [70,71,72]. Therefore, if the miRNA binding site located within the retained intron is also present in the 5′ or 3′ UTR of the main transcript, both variants could be targeted by the same miRNA [70,71,72]. This is the reason why it became feasible to use the TarBase tool to identify DAPK-1-related microRNAs that could also modulate s-DAPK-1. The TarBase tool has also been used by other researchers, deeming it a valuable tool in the literature for predicting microRNAs targeting different genes [73,74]. In line with the objective of this study, the TarBase tool predicted that hsa-miR-30a-5p and hsa-miR-26a-5p could target mitogen-activated protein kinase 10 (*MAPK10*), protein phosphatase, Mg^2+^/Mn^2+^-dependent 1J (*PPM1J*), and RB1 inducible coiled-coil 1 (*RB1CC1*) autophagy-related genes [73]. Additionally, this tool effectively predicted connective tissue growth factor (*CTGF*) and periostin (*POSTN*) as direct targets of miR-30a-5p in heart failure conditions [74].

MiRNAs primarily operate by binding to the 3′ untranslated region (UTR) on the messenger RNA post-transcription, thus suppressing the expression of their gene targets, thereby, controlling a wide range of biological and pathological processes, such as the initiation and progression of cancer [75,76]. The scientific literature has extensively documented miR-26a-5p and miR-26b-5p identified in Table 1 as potential regulators of s-DAPK-1 for their tumor-suppressing activities in hepatocellular carcinoma and gastric cancer, respectively [42,43,44]. Therefore, their potential binding to s-DAPK-1 mRNA will influence its translation and function.

Given that a protein’s function is related to its structure [77,78,79], upon discovering the potential of microRNAs to regulate s-DAPK-1, curiosity led us to explore the three-dimensional structure of this protein. Firstly, the prediction of s-DAPK-1’s secondary structures was conducted, and the results showed significant prevalence of helical configurations linked by coils; these helix-turn-helix motifs detected in s-DAPK-1’s secondary structure primarily function in DNA binding and sequence-specific recognition, often acting as transcription factors to modulate gene expression [80,81]. This implies that s-DAPK-1 is likely to be involved in some or all these biological processes as predicted in Table 4a.

The helical configurations dominating s-DAPK-1 initially suggested that s-DAPK-1 may be classified as a globular protein, because most globular proteins have been reported to have at least 30% alpha helices in their globular structure [82]. However, further analysis of the data showed that the detected alpha helices are succeeded by coils. These secondary structures can sequentially arrange themselves, giving rise to repeated sequence motifs [83], which might result in a long, elongated tertiary structure that differs significantly from the compact and spherical forms typically seen in globular proteins.

Indeed, the 3D structure of s-DAPK-1 modeled using I-TASSER proved that s-DAPK-1 does not resemble the characteristics of a globular protein because it is made of two antiparallel α-helices and a β-loop, which are packed together to form a helix-turn-helix bundle known as ankyrin repeats. I-TASSER was used to model the 3D structure of s-DAPK-1 because it has been proven to generate models that closely match experimental ones. A study conducted by Kemege et al. [84] serves as an example, where I-TASSER was employed to model the CT296 structure. Subsequently, the structure was experimentally resolved using X-ray crystallography [84]. Impressively, the model generated by I-TASSER exhibited a close match (RMSD: 2.72 Å) to the high-resolution (1.8 Å) crystal structure of CT296. As per the findings from the I-TASSER webserver, the modeled structure of s-DAPK-1 prominently featured ankyrin repeats, mirroring the ankyrin repeats identified in the DAPK-1 structure [10]. It is noteworthy that these ankyrin repeats in both isoforms consist of the same amino acids. Ankyrin repeats have been identified in a wide range of proteins, serving various functions, such as cell-to-cell communication, maintenance of the cytoskeleton structure, regulation of transcription and cell cycle, inflammatory response, development, and diverse transport processes [83,85,86]. Interestingly, some of these activities are predicted to be the functions of s-DAPK-1 (Table 4a).

Prediction of s-DAPK-1’s 3D structure enabled us to predict its physical, chemical, and thermodynamic parameters. The physicochemical properties revealed that s-DAPK-1 is an unstable protein. This is not surprising as Lin et al. [10] demonstrated that s-DAPK-1′s instability is a consequence of its unique tail that is not found in DAPK-1. This tail is responsible for regulating the protein’s localization and stability, and can be cleaved by proteases within cells. Similar to DAPK-1, s-DAPK-1 demonstrates a more precise localization within the cytoplasm. Notably, when the tail of s-DAPK-1 is deleted, it predominantly localizes in the vicinity of the nucleus, ultimately enhancing its stability and membrane blebbing activity [10,87]. Nevertheless, the cleavage of the tail region of s-DAPK-1 in vivo is significantly diminished when s-DAPK-1 is inserted into GFP vectors and expressed in cells. This observation indicates that protein tags possess the ability to alter the conformational structures of their fusion proteins [88,89].

When observing the predicted 3D structure of s-DAPK-1, it is evident that the s-DAPK-1 structure is dominated by ankyrin repeats that play a major role in mediating protein–protein interactions [83,85,90]. Therefore, it is not surprising to see s-DAPK-1 interacting with other proteins as presented in Table 3. These interactions potentially play a crucial role in enabling s-DAPK-1 to carry out diverse functions, as depicted in Table 4a, because for a protein to effectively perform its designated function, it typically needs to interact with various other molecules, and this interaction is directly influenced by its 3D structure [91]. This is feasible since most proteins, although capable of independent function, often rely on interactions with other proteins for optimal biological activity [92]. Among the proteins that may interact with s-DAPK-1 (Table 3), H2B2E stands out due to its association with nucleosomes. Nucleosomes play a crucial role in regulating the accessibility of the transcription machinery to DNA and serve as a fundamental component in the intricate regulation of gene expression [93]. In the realm of apoptosis, it has been widely proposed that histone modifications play a significant role in influencing the function and structure of chromatin during cell death [94]. Considering the possible affinity of s-DAPK-1 towards H2B2E, it is plausible to suggest that epigenetic alterations of H2B2E may also contribute to the initiation of apoptosis, which is one of the functions of DAPK-1. Consequently, if s-DAPK-1 is capable of inducing apoptosis through the epigenetic modulation of H2B2E, it can be inferred that both s-DAPK-1 and DAPK-1 share the common function of promoting apoptosis.

In summary, while experimental validation (“wet lab experiments”) remain essential for confirming bioinformatics-derived findings, such data retain considerable value in their own right. Specifically, we envision that the insights generated from these bioinformatics analyses can drive renewed interest in s-DAPK-1 and inform the development of more experimental designs and optimization strategies, particularly for the investigation of less-studied proteins, such as s-DAPK-1. This is further supported by numerous studies in which researchers employed exclusively in silico approaches to study protein structure and function, underscoring the growing credibility and utility of advanced computational methods in molecular biology [95,96,97]. For example, Arshad et al. [98] used the Phyre2 and I-TASSER homology modeling tools to generate 3D models of wild-type T-cell activation RhoGTPase-activating protein (TAGAP) and TAGAP mutated with high-risk single nucleotide polymorphisms (SNPs). The research conducted by Khan et al. [99] also relied entirely on in silico data to predict the structure and function of *S. typhi* hypothetical protein (HP) R_27 using only the protein sequence as a basis. Clearly, investigations to understand protein structure and function based solely on in silico data continue to gain momentum [95,96,97].

## 5. Conclusions

In an effort to gain a more comprehensive understanding of the biological complexity of s-DAPK-1, bioinformatics tools facilitated the prediction of s-DAPK-1’s physical, chemical, and thermodynamic parameters, 3D structure, functionality, and protein–protein interactions. The findings of this study lay the groundwork for future studies aimed at discovering the potential drug targets associated with s-DAPK-1, regulating its expression, and exploring its various functions. Moreover, given that s-DAPK-1 is less studied, these computational results are likely to stimulate interest in s-DAPK-1, thus fostering ongoing studies on s-DAPK-1 to solidify the in silico findings.

## Figures and Tables

**Table 1:** Predicted microRNAs targeting s-DAPK-1 mRNA.

Predicted microRNA	Function	Binding Site	References
hsa-miR-26a-5p	Tumor suppressor in hepatocellular carcinoma and lung cancer	5′ UTR	[41,42,43]
hsa-miR-26b-5p	Tumor suppressor in gastric cancer	5′ UTR	[44]

**Table 2:** Physicochemical properties of s-DAPK-1.

Parameters	Values
Number of amino acids	337
Molecular weight (Da)	36,739.07
Theoretical pI	8.96
Extinction coefficients	27,680 ^a^ 26,930 ^b^
Half-life	30 h (mammalian reticulocytes, in vitro) >20 h (yeast, in vivo) >10 h (*Escherichia coli*, in vivo)
Instability index	40.67
Aliphatic index	87.74
Grand average of hydropathicity (GRAVY)	−0.221

Note: ^a^ value predicted on the basis that all pairs of Cys residues form cystines; ^b^ value predicted on the basis that all Cys residues are reduced.

**Table 3:** Proteins predicted to interact with s-DAPK-1.

Protein Name	Uniprot ID	Score (1 = Binding)	Function
Keratin-associated protein 4–12	Q9BQ66	0.8173	Important for making hair strong, this happens when disulfide bonds connect cysteines of hair keratins [45,46]
Histone H2B type 2-E	Q16778	0.7173	Responsible for wrapping and compacting DNA into chromatin, thus limiting DNA accessibility to cellular machinery that uses DNA as a template [47,48]
Prion protein	Q53YK7	0.7093	Promotes tumor progression [49]
Nuclear protein 1	O60356	0.7107	Promotes cancer progression [50]
Dynein light chain 1, cytoplasmic	P63167	0.7200	Regulates apoptosis by sequestering BCL2L11 in microtubules [51,52]
Microtubule-associated protein 1 light chain 3 beta, isoform CRA_c	Q658J6	0.7200	Regulates autophagy [53]
Microtubule-associated proteins 1A/1B light chain 3B	Q9GZQ8	0.7200	Involved in the formation of autophagosomes [54]
Polyubiquitin-B	P0CG47	0.7133	Regulates protein ubiquitination [55,56]
Epididymis secretory protein Li 50	Q5U5U6	0.7133	Targets cellular proteins for degradation by the 26S proteosome [57]
Prenylated Rab acceptor protein 1	Q9UI14	0.7133	Necessary for the vesicle formation from the Golgi complex [58,59]

**Table 4:** (**a**) Predicted functions of s-DAPK-1. (**b**) Predicted functions of DAPK-1.

**(a)**			
**Biological Process Predictions**			
**GO Term**	**Name**	**Probability Score**	**SVM Reliability**
GO:1903506	regulation of nucleic acid-templated transcription	0.829	H
GO:2001141	regulation of RNA biosynthetic process	0.813	H
GO:0051252	regulation of RNA metabolic process	0.808	H
GO:0051171	regulation of nitrogen compound metabolic process	0.769	H
GO:0034645	cellular macromolecule biosynthetic process	0.767	H
GO:0006355	regulation of transcription, DNA-templated	0.760	H
GO:0009116	nucleoside metabolic process	0.719	H
GO:0006810	transport	0.718	H
GO:0019222	regulation of metabolic process	0.710	H
GO:0006796	phosphate-containing compound metabolic process	0.704	H
GO:0055086	nucleobase-containing small molecule metabolic process	0.684	H
GO:0044281	small molecule metabolic process	0.675	H
GO:0009059	macromolecule biosynthetic process	0.646	H
GO:0010468	regulation of gene expression	0.638	H
GO:0006163	purine nucleotide metabolic process	0.634	H
GO:0045184	establishment of protein localization	0.608	H
GO:0019637	organophosphate metabolic process	0.605	H
GO:0045333	cellular respiration	0.577	H
GO:0051641	cellular localization	0.571	H
GO:0006091	generation of precursor metabolites and energy	0.551	H
GO:0055114	oxidation-reduction process	0.547	H
GO:0009259	ribonucleotide metabolic process	0.534	H
GO:0051649	establishment of localization in cell	0.531	H
GO:0006082	organic acid metabolic process	0.523	H
GO:0006412	translation	0.510	H
**Molecular function predictions**			
GO:0008270	zinc ion binding	0.932	H
GO:0003723	RNA binding	0.754	H
GO:0003824	catalytic activity	0.715	H
GO:0015631	tubulin binding	0.713	H
GO:0003676	nucleic acid binding	0.708	H
GO:0000166	nucleotide binding	0.687	H
GO:0035639	purine ribonucleoside triphosphate binding	0.679	H
GO:0017076	purine nucleotide binding	0.669	H
GO:0001883	purine nucleoside binding	0.659	H
GO:0008092	cytoskeletal protein binding	0.658	H
GO:0016491	oxidoreductase activity	0.611	H
GO:0001882	nucleoside binding	0.609	H
GO:0032549	ribonucleoside binding	0.601	H
GO:0030554	adenyl nucleotide binding	0.552	H
GO:0003779	actin binding	0.541	H
GO:0016740	transferase activity	0.533	H
GO:0019901	protein kinase binding	0.530	H
GO:0003677	DNA binding	0.520	H
GO:0046872	metal ion binding	0.741	L
GO:0005102	receptor binding	0.691	L
GO:0036094	small molecule binding	0.666	L
GO:0043169	cation binding	0.595	L
GO:0032403	protein complex binding	0.554	L
GO:0019904	protein domain specific binding	0.539	L
**(b)**			
**Biological Process Predictions**			
**GO Term**	**Name**	**Probability Score**	**SVM Reliability**
GO:0006796	phosphate-containing compound metabolic process	0.813	H
GO:0008380	RNA splicing	0.807	H
GO:0006811	ion transport	0.753	H
GO:0019222	regulation of metabolic process	0.735	H
GO:0006810	transport	0.672	H
GO:0016310	phosphorylation	0.648	H
GO:0000398	mRNA splicing, via spliceosome	0.638	H
GO:0044281	small molecule metabolic process	0.614	H
GO:0051171	regulation of nitrogen compound metabolic process	0.598	H
GO:0006396	RNA processing	0.566	H
GO:0019637	organophosphate metabolic process	0.557	H
GO:1903506	regulation of nucleic acid-templated transcription	0.552	H
GO:2001141	regulation of RNA biosynthetic process	0.551	H
GO:0009059	macromolecule biosynthetic process	0.546	H
GO:0006397	mRNA processing	0.539	H
GO:0071345	cellular response to cytokine stimulus	0.527	H
GO:0006082	organic acid metabolic process	0.511	H
GO:0055086	nucleobase-containing small molecule metabolic process	0.510	H
GO:0051252	regulation of RNA metabolic process	0.510	H
GO:0006355	regulation of transcription, DNA-templated	0.505	H
GO:0055114	oxidation-reduction process	0.503	H
GO:0008152	metabolic process	0.925	L
GO:0050896	response to stimulus	0.869	L
GO:0051716	cellular response to stimulus	0.836	L
GO:0044237	cellular metabolic process	0.791	L
**Molecular Function Predictions**			
GO:0003824	catalytic activity	0.973	H
GO:0035639	purine ribonucleoside triphosphate binding	0.939	H
GO:0017076	purine nucleotide binding	0.938	H
GO:0030554	adenyl nucleotide binding	0.925	H
GO:0032549	ribonucleoside binding	0.921	H
GO:0000166	nucleotide binding	0.914	H
GO:0001883	purine nucleoside binding	0.911	H
GO:0005524	ATP binding	0.899	H
GO:0001882	nucleoside binding	0.844	H
GO:0016817	hydrolase activity, acting on acid anhydrides	0.766	H
GO:0016773	phosphotransferase activity, alcohol group as acceptor	0.759	H
GO:0044822	poly(A) RNA binding	0.758	H
GO:0016301	kinase activity	0.751	H
GO:0017111	nucleoside-triphosphatase activity	0.740	H
GO:0004386	helicase activity	0.666	H
GO:0003676	nucleic acid binding	0.650	H
GO:0016462	pyrophosphatase activity	0.636	H
GO:0003723	RNA binding	0.633	H
GO:0016818	hydrolase activity, acting on acid anhydrides, in phosphorus-containing anhydrides	0.596	H
GO:0016740	transferase activity	0.593	H
GO:0016491	oxidoreductase activity	0.523	H
GO:0008092	cytoskeletal protein binding	0.517	H
GO:0046872	metal ion binding	0.887	L
GO:0097159	organic cyclic compound binding	0.869	L
GO:0016772	transferase activity, transferring phosphorus-containing groups	0.862	L

## Data Availability

All data generated or analyzed throughout this study are contained in this article and the Supplementary Information Files.

## Supplementary Materials

The following supporting information can be downloaded at: https://www.mdpi.com/article/10.3390/cimb47060416/s1.
