# Supplementary material for: In Silico Analysis of s-DAPK-1: From Structure to Function and Regulation
Source: Curr Issues Mol Biol. 2025 Jun 4;47(6):416. doi: 10.3390/cimb47060416 (PMC12192072; doi:10.3390/cimb47060416)
Supplement: Supplementary file 1 [file cimb-47-00416-s001.zip › cimb-3622427-supplementary.pdf]

Supplementary S1

Gene name	miRNA name	Experiments	Publications	Cell lines	microT Score	Details
DAPK1	hsa-miR-26a-5p	7	5	4	0.95	
DAPK1	hsa-miR-26b-5p	5	3	2	0.95	
DAPK1	hsa-miR-154-3p	3	3	2	0.83	
DAPK1	hsa-miR-487a-3p	2	2	1	0.91	

Figure S1. MicroRNAs targeting DAPK-1. Symbol "i" is supplemental information about gene and miRNA details, the downward facing arrows are used to expand specific interactions and to reveal metainformation.

Supplementary S2

hsa-miR-26a-5p sequence: UUCAAGUAAUCCAGGAUAGGCU

hsa-miR-26b-5p sequence: UUCAAGUAAUUCAGGAUAGGU

```
CLUSTAL multiple sequence alignment by MUSCLE (3.8)


DAPK-1          ----GCTTCGGAGTGTGAGGAGGACAGCCGGACCGAG---------CCAA-------CGC
s-DAPK-1        ATTTACTCAACAATGTTAGATGTCACACATGAAAAAGTTTTGTTATCTAATTATTCTCAT
                     **    * *** **  *     *  **   **         * **       *

DAPK-1          CGGGGACTTTGTTCCCTCC--------------------------GCGGAGGGGACTCGG
s-DAPK-1        CAGAAA-TTTGTTGCCTGCTTCTGCAGACGTCAGCAGAGCTGTAAGTGGAGTAGATCTGG
                * *  * ****** *** *                          * ****  **   **

DAPK-1          CAACTCGCAGCGGCAGGGTCTGGGGCCGGCGCCTGGGAGGGATCTGCGCCCCC-------
s-DAPK-1        CCCTCTGCAGTATGGGAATCC-TAACCAGAG---AGGGGAGATGAGGGTTTACAAACATA
                *     ****     *  **     ** * *    ** * ***  * *    *

DAPK-1          --------------------------------CACTCACTCCCTAGCT------------
s-DAPK-1        AAATAATTGTTTATAAGCAATTGTTTATTTTGAAATCATTTTTGATTTACAAAAAAAAGT
                                                 * *** *    *  *

DAPK-1          -----------------GTGTTCCCGCCGCCGCCCCGGCTA---GTCTCCGGCGCTGGCG
s-DAPK-1        TGGAAAAGTGGTACAGAGAGTTCCATCATTCGCTTCACCCAGGTGCCCCTAATGCTAGCA
                                 * *****  *   ***  *  * *   * * *    *** **

DAPK-1          CCT---------ATGGTCGGCCTCCGACAGCGCTCCGGAG-GGACCGGGGGAGCTCCCAG
s-DAPK-1        TCTTGTGTAACCACGGT--ACATTTGTCAAAGGTGTAAAATAGAATGAGGG---TTTTAT
                 **         * ***   * *  * **  * *    *   **  * ***   *   *

DAPK-1          GCGCCCGGGACTGGAGACTGATGCATGAGGGGGCTACGGAGGCGCAGGAGCGGTGGTGAT
s-DAPK-1        TCCTCTGGGA------AGTTGCTCCTCTGGGGTTTGTCATCACACAGGAAGGGAAATAAC
                 *  * ****      * *    * *  ****  *       * *****  **   * *

DAPK-1          ----GGTCTGGGAA----GCGGAGCTGAAGTGCCCTGGGCTTTGGTGAGGCGTGAC----
s-DAPK-1        AGTGAGTCTGAGGAATGCATGCATCTG--GTGCTGTCAGCTCCCTAGAAATGTGACCCCA
                     ***** * *      * * ***  ****  *  ***     **   *****

DAPK-1          AGTTTATCAT------GACCGTGTTCAGGCAGGAAAACGTGGATGATTACTACGACACCG
s-DAPK-1        GGTTGATTTTCCTGCAGGGCGGGTCCAATGCCGTCTACTGGGCTGCTCGGCATGGCCACG
                 *** **  *      *  ** ** **     *   **  ** ** *    * * *  **

DAPK-1          GCGA------GGAACTTGGCAGTGGACAGTTTGCGGTTGTGAAGAAATGCC------GTG
s-DAPK-1        TCGATACCTTGAAATTTCTCAGTGA---------------GAACAAATGCCCTTTGGATG
                 ***      * ** **  *****                *** *******       **

DAPK-1          AGAAAAGCACCGGCCTCCAGTATGCCGCCAAATTCATCAAGAAAAGGAGG----ACTAAG
s-DAPK-1        TGAAAGACAAGGTA-------AGGCCACTTCTCTTAGGAGGAACATGAGGTGGTAGTAAA
                 ****  **  *         * *** *     * *  * *** * ****    * ***
```

```
DAPK-1          TCCAGCCGGCGGGGTGTGAG----------CCGCGAGGACATCGAGC-------GGGAGG
s-DAPK-1        TGGATGCATGTGAGTGTGAGTGCTGGCCTACCGTGTG--CATCGGGACCCAAAGGAAAGG
                *  *  *    * *******          *** * *  ***** *        *  ***

DAPK-1          T--------CAGCAT---CCTGAAGGAGATCCAGCACCCCAATGTCATCACCCTGCACGA
s-DAPK-1        TATCAGAACCAGAATTCACCTGCAGAACATTTAAGTTGGAAATGTCTT---GTTGCCTGT
                *        *** **   **** ** * **  *       ****** *     ***  *

DAPK-1          GGTCTATGAGAACAAGACGGACGTCATCCTGATCTTGGAACTCGTTGCAGGTGG------
s-DAPK-1        GGTTT---GGTCTAATACTGAAATCAGCCTTAAGTCAAAATC--TTTCAGGTAGTTGCCA
                *** *    *   ** ** **  *** *** *  *   **    ** ***** *

DAPK-1          -----CGAGCTGTTTGACTT--------CTTAGCTGAAAAG----GAATCTTT-------
s-DAPK-1        TAATACGCACGCATTGAATTATAATCCCCATAACATAAAAACCTCAAAGTTCTAGTACAG
                     **  *   **** **        * ** *  ****      **  * *

DAPK-1          --AACTGAAGAGGAA--------GCAACTGAATTT---------CTCAAACAAATTCTTA
s-DAPK-1        ACAACCAGAGAGAGACCTCGGTCTCGATTCGATTTTCTTCCTGACTCAACCTTGTGCTTT
                  ***   ****  *         * * *  ****         ***** *   * ***

DAPK-1          ATGGTGTTTACTACCTGCACTC-----CCTTCAAATCGCCCACTTTG-----------AT
s-DAPK-1        TAAGCGGATCTCCCCTATTCTCTTCTTCTTTGAAGTGGCTGACAGTGCTGACCTGACAAT
                   * *  *    ***   ***     * ** ** * **  **  **           **

DAPK-1          CTTAAGCCTGAGAACATAATGCTTTTGGATAGA-AATGTCCCCAAACCTCGGATCAAGAT
s-DAPK-1        CACAGACACTAGAAGATA------TTAATGAGACAATGCACATAAA----GTAATAAAGT
                *  *  *   **** ***      **    *** ****  *  ***    * *  **  *

DAPK-1          CATTGACTTTGGGTTGGCCC---------ATAAAATTGACTTTGGAAATGAATTTAAAAA
s-DAPK-1        AA--GGCTTTTTTTTGGCACAACGGTGTTATAAAAGTTACT---------GATTTATCAA
                 *  * ****   ***** *         ****** * ***          *****  **

DAPK-1          CATATTTGGGACTCCAGAGTTTGT---------CGCTCCTGAGATAGTCAACTATGAACC
s-DAPK-1        GATACCTTGTTTTCATATGTGTGTGTTTCATTCAGTTCACACAACAATC--CTGTGAATT
                 ***  * *   **    ** ***          * **     * * **  ** ****

DAPK-1          TCTTGGT----CTTGAGGCAGATATGTGGAGTATCGGGGTAATAACCTA-----TATCCT
s-DAPK-1        AATTGGCTGTGCTGGAACCTGA-ACCTGGAATTCCTACCTACTCATCCATTATTTATTCT
                  ****     ** **  * ** *  **** *  *    ** * * * *     *** **

DAPK-1          CCTAAGTGGGGCCTCCCCATTTCTTGGA---------------GACACTAAG-CAAGAAA
s-DAPK-1        ACTGTACAATACTTCTCTGCTTCCAGTACTGGCAGACTAATTTGACGTTTAGTCAAGCTG
                 **        * ** *   ***  * *               ***  * ** ****

DAPK-1          CGTTA-GCAAATGTATCCGCTGTCAACTACGA---ATTTGAGGATGAATACT----TCAG
s-DAPK-1        CCACATGCAGTTGCATGGGTTGTGCACTGCACCATATCTGAGGGGTAACATTCATATCAG
                *   * ***  ** **  * ***  *** *     ** *****   ** * *    ****
```

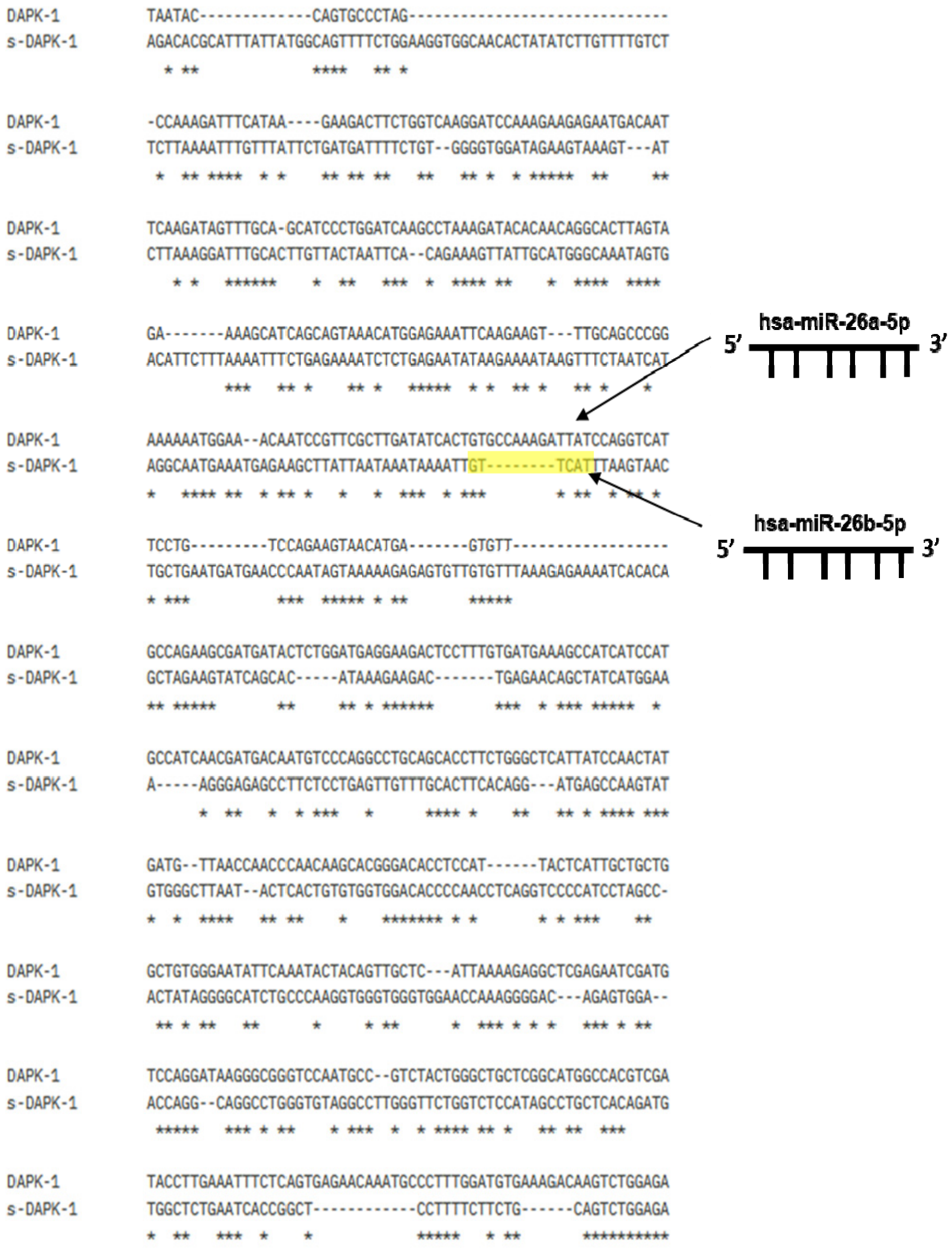
DAPK-1
s-DAPK-1
hsa-miR-26a-5p
5'
3'
hsa-miR-26b-5p
5'
3'

```
DAPK-1          GATGGCCCTCCACGTGGCAGCTCGCTATGGCCATGCTGACGTGGCTCAGTTACTGTGCAG
s-DAPK-1        GATGGCCCTCCACGTGGCAGCTCGCTATGGCCATGCTGACGTGGCTCAGTTACTGTGCAG
                ************************************************************

DAPK-1          CTTCGGCTCAAATCCCAATATCCAGGACAAGGAAGAAGAAACCCCCCTGCACTGTGCTGC
s-DAPK-1        CTTCGGCTCAAATCCCAATATCCAGGACAAGGAAGAAGAAACCCCCCTGCACTGTGCTGC
                ************************************************************

DAPK-1          TTGGCACGGCTATTACTCTGTGGCCAAAGCCCTTTGTGAAGCCGGCTGTAACGTGAACAT
s-DAPK-1        TTGGCACGGCCATTACTCTGTGGCCAAAGCCCTTTGTGAAGCCGGCTGTAACGTGAACAT
                ********** *************************************************

DAPK-1          CAAGAACCGAGAAGGAGAGACGCCCCTCCTGACAGCCTCTGCCAGGGGCTACCACGACAT
s-DAPK-1        CAAGAACCGAGAAGGAGAGACGCCCCTCCTGACAGCCTCTGCCAGGGGCTACCACGACAT
                ************************************************************

DAPK-1          CGTGGAGTGTCTGGCCGAACATGGAGCCGACCTTAATGCTTGCGACAAGGACGGACACAT
s-DAPK-1        CGTGGAGTGTCTGGCCGAACATGGAGCCGATCTTAATGCTTGCGACAAGGACGGACACAT
                ****************************** *****************************

DAPK-1          TGCCCTTCATCTGGCTGTAAGACGGTGTCAGATGGAGGTAATCAAGACTCTCCTCAGCCA
s-DAPK-1        TGCCCTTCATCTGGCTGTAAGACGGTGTCAGATGGAGGTAATCAAGACTCTCCTCAGCCA
                ************************************************************

DAPK-1          AGGGTGTTTCGTCGATTATCAAGACAGGCACGGCAATACTCCCCTCCATGTGGCATGTAA
s-DAPK-1        AGGGTGTTTCGTCGATTATCAAGACAGGCACGGCAATACTCCCCTCCATGTGGCATGTAA
                ************************************************************

DAPK-1          AGATGGCAACATGCCTATCGTGGTGGCCCTCTGTGAAGCAAACTGCAATTTGGACATCTC
s-DAPK-1        AGATGGCAACATGCCTATCGTGGTGGCCCTCTGTGAAGCAAACTGCAATTTGGACATCTC
                ************************************************************

DAPK-1          CAACAAGTATGGGCGAACGCCTCTGCACCTTGCGGCCAACAACGGAATCCTAGACGTGGT
s-DAPK-1        CAACAAGTATGGGCGAACGCCTCTGCACCTTGCGGCCAACAACGGAATCCTAGACGTGGT
                ************************************************************

DAPK-1          CCGGTATCTCTGTCTGATGGGAGCCAGCGTTGAGGCGCTGACCACGGACGGAAAGACGGC
s-DAPK-1        CCGGTATCTCTGTCTGATGGGAGCCAGCGTTGAGGCGCTGACCACGGACGGAAAGACGGC
                ************************************************************

DAPK-1          AGAAGATCTTGCTAGATCGGAACAGCACGAGCACGTAGCAGGTCTCCTTGCAAGACTTCG
s-DAPK-1        AGAAGATCTTGCTAGATCGGAACAGCACGAGCACGTAGCAGGTCTCCTTGCAAGACTTCG
                ************************************************************

DAPK-1          AAAGGATACGCACCGAGGACTCTTCATCCAGCAGCTCCGACCCACACAGAACCTGCAGCC
s-DAPK-1        AAAGGATACGCACCGAGGACTCTTCATCCAGCAGCTCCGACCCACACAGAACCTGCAGCC
                ************************************************************
```

```
DAPK-1          AAGAATTAAGCTCAAGCTGTTTGGCCACTCGGGATCCGGGAAAACCACCCTTGTAGAATC
s-DAPK-1        AAGAATTAAGCTCAAGCTGTTTGGCCACTCGGGATCCGGGAAAACCACCCTTGTAGAATC
                ************************************************************

DAPK-1          TCTCAAGTGTGGGCTGCTGAGGAGCTTTTTCAGAAGGCGTCGGCCCAGACTGTCTTCCAC
s-DAPK-1        TCTCAAGTGTGGGCTGCTGAGGAGCTTTTTCAGAAGGCGTCGGCCCAGACTGTCTTCCAC
                ************************************************************

DAPK-1          CAACTCCAGCAGGTTCCCACCTTCACCCCTGGCTTCTAAGCCCACAGTCTCAGTGAGCAT
s-DAPK-1        CAACTCCAGCAGGTTCCCACCTTCACCCCTGGCTTCTAAGCCCACAG-----GTAGG---
                ***********************************************     ** *

DAPK-1          CAACAACCTGTACCCAGGCTGCGAGAACGTGAGTGTGAGGAGCCGCAGCATGATGTTCGA
s-DAPK-1        ----AACCT----CCATGCTG---------------------------------------
                    *****    *** ****

DAPK-1          GCCGGGTCTTACCAAAGGGATGCTGGAGGTGTTTGTGGCCCCGACCCACCACCCGCACTG
s-DAPK-1        GCCCCGTCTCTCCA----------GCAGGTGTTGG--------------CTTCCGCACT-
                ***  ****  ***          * ******* *              *  *******

DAPK-1          CTCGGCCGATGACCAGTCCACCAAGGCCATCGACATCCAGAACGCTTATTTGAATGGAGT
s-DAPK-1        ------------CTCTCCTTTCAAGG---------TCTAGGGG--------GAAGGGAGT
                            *    *   *****         ** **  *        *** *****

DAPK-1          TGGCGATTTCAGCGTGTGGGAGTTCTCTGGAAATCCTGTGTATTTCTGCTGTTATGACTA
s-DAPK-1        TG-----------------------------------------------TGTTTGGGTCA
                **                                               ****  *   *

DAPK-1          TTTTGCTGCAAATGATCCCACGTCAATCCATGTTGTTGTCTTTAGTCTAGAAGAGCCCTA
s-DAPK-1        CTTGGCCTATACTGGACCC--------------TGTGGCCTT------------------
                 ** **    * **  ***              *** * ***

DAPK-1          TGAGATCCAGCTGAACCAAGTGATTTTCTGGCTCAGTTTCCTGAAGTCCCTTGTCCCAGT
s-DAPK-1        ------------------AGTGGTTTTCAGGTCCAGGTT---------------------
                                  **** ***** **  *** **

DAPK-1          TGAAGAACCCATAGCCTTCGGTGGCAAGCTGAAGAACCCACTCCAAGTTGTCCTGGTGGC
s-DAPK-1        -----GGCTGGTAGCCTTGTGTG---------------TGCTGCCTGTTGTCCAG-----
                       *   *******  ***                 ** *  ******* *

DAPK-1          CACCCACGCTGACATCATGAATGTTCCTCGACCGGCTGGAGGCGAGTTTGGATATGACAA
s-DAPK-1        ------CAGTAACA---------------GACTGAC-----------TTGGCAGTAGCAA
                      *  * ***               *** * *           ****   *  ***

DAPK-1          AGACACATCGTTGCTGAAAGAGATTAGGAACAGGTTTGGAAATGATCTTCACATTTCAAA
s-DAPK-1        AACAAGGGCCTCACTGAAA-----------------------------TCACA-------
                *   *   * *  ******                             *****
```

```
DAPK-1          TAAGCTGTTTGTTCTGGATGCTGGGGCTTCTGGGTCAAAGGACATGAAGGTACTTCGAAA
s-DAPK-1        ---------------------------------GCCACA-GACACAAAGGTCCATC----
                                                 * ** * ****  ***** * **

DAPK-1          TCATCTGCAAGAAATACGAAGCCAGATTGTTTCGGTCTGTCCTCCCATGACTCACCTGTG
s-DAPK-1        --------------------------------------------------CTCATAAATG
                                                                  ****    **

DAPK-1          TGAGAAAATCATCTCCACGCTGCCTTCCTGGAGGAAGCTCAATGGACCCAACCAGC--TG
s-DAPK-1        TGCAAA---------------------CTGCAG-------AATTTGCACAGTCAGCTTTG
                **  **                     *** **       ***   * **  ****  **

DAPK-1          ATGTCGCTGCAGCAGTTTGTGTACGACGTGCAGGACCAGCTGAACCCCCTGGCCAGCGAG
s-DAPK-1        GGGACACAGTTGCAAATTGCAT------TGCA--------------TTCTACTCAGGG--
                  * * * *  ***  ***  *      ****                **   *** *

DAPK-1          GAGGACCTCAGGCGCATTGCTCAGCAGCTCCACAGCACAGGCGAGATCAACATCATGCAA
s-DAPK-1        ------CTCTGGC------CTTA-------------------GAGACAAAAAGAGCTCAA
                      *** ***      ** *                   ****  ** *     ***

DAPK-1          AGTGAAACAGTTCAGGACGTGCTGCTCCTGGACCCCCGCTGGCTCTGCACAAACGTCCTG
s-DAPK-1        ACTCAAACAGTCCA--------------------------AGCATAGCACAGA--TCC--
                * * ******* **                           **   ***** *  ***

DAPK-1          GGGAAGTTGCTGTCCGTGGAGACCCCACGGGCGCTGCACCACTACCGGGGCCGCTACACC
s-DAPK-1        ----AATAGCAGCCCAAGAAGGGTCCTCAACCCTTATCCTTCTTTCTGGAGTGCTACA--
                    * * ** * **  * **   ** *   *  *   *  **  * **   ******

DAPK-1          GTGGAGGACATCCAGCGCCTGGTGCCCGACAGCGACGTGGAGGAGCTGCTGCAGATCCTC
s-DAPK-1        ----GGCAGACCCAG-------------------------------TATTGTAGATACAT
                     * * * ****                               *  ** **** *

DAPK-1          GATGCCATGGACATCTGCGCCCGGGACCTGAGCAGCGGGACCATGGTGGACGTCCCAGCC
s-DAPK-1        AGAGCAACAAAAA---------GGGATTT-----------CCAGGAAGCACCATCCTGCT
                   ** *   * *         ****  *           *** *  * **   ** **

DAPK-1          CTGATCAAGACAGACAACCTGCACCGCTCCTGGGCTGATGAGGAGGACGAGGTGATGGTG
s-DAPK-1        CAGTTCAAG-----------------------------------------------GTTG
                * * *****                                               * **

DAPK-1          TATGGTGGCGTGCGCATCGTGCCCGTGGAACACCTCACCCCCTTCCCATGTGGCATCTTT
s-DAPK-1        GATAGAGG------------------------------------------------TTTC
                 ** * **                                                 **

DAPK-1          CACAAGGTCCAGGTGAACCTGTGCCGGTGGATCCACCAGCAAAGCACAGAGGGCGACGCG
s-DAPK-1        CAGAGGCTCC--------------------------------------------------
                ** * * ***
```

```
DAPK-1          GACATCCGCCTGTGGGTGAATGGCTGCAAGCTGGCCAACCGTGGGGCCGAGCTGCTGGTG
s-DAPK-1        ----------------TTAGTGGTTTTCAG----------------------GGATGGTC
                                * * *** *   **                       * ****

DAPK-1          CTGCTGGTCAACCACGGCCAGGGCATTGAGGTCCAGGTCCGCGGCCTGGAGACGGAGAAG
s-DAPK-1        TCTCTGGT----CACATCCA-------------------CCCTCCTTGGGAACGT-----
                   *****    ***  ***                   * *  * ***  ***

DAPK-1          ATCAAGTGCTGCCTGCTGCTGGACTCGGTGTGCAGCACCATTGAGAACGTCATGGCCACC
s-DAPK-1        -----TTGCTGCCTTCCG-----------------------------------GGCCTTC
                      ******** * *                                   ****  *

DAPK-1          ACGCTGCCAGGGCTCCTGACCGTGAAGCATTACCTGAGCCCCCAGCAGCTGCGGGAGCAC
s-DAPK-1        -CACAGACAGGGTTATTGATT----------------------------------CACAT
                 * * * ***** *  ***                                      **

DAPK-1          CATGAGCCCGTCATGATCTACCAGCCACGGGACTTCTTCCGGGCACAGACTCTGAAGGAA
s-DAPK-1        TAGGGGGCTGTCACGA--------------------------------------------
                 * * * * **** **

DAPK-1          ACCTCACTGACCAACACCATGGGGGGGTACAAGGAAAGCTTCAGCAGCATCATGTGCTTC
s-DAPK-1        ------------AACACC-TGGCAGTGAGCAAGAGAAAATGATGCA---------ATCTT
                            ****** ***  * *  ****  **  *   ***            *

DAPK-1          GGGTGTCACGACGTCTACTCACAGGCCAGCCTCGGCATGGACATCCATGCATCAGACCTG
s-DAPK-1        GGGTGTGA----------------------------------------------------
                ****** *

DAPK-1          AACCTCCTCACTCGGAGGAAACTGAGTCGCCTGCTGGACCCGCCCGACCCCCTGGGGAAG
s-DAPK-1        ---TTCC------------AACTGAATC-------------------CTCCTTTAGACAG
                    ***            ****** **                   * ** *  *  **

DAPK-1          GACTGGTGCCTTCTCGCCATGAACTTAGGCCTCCCTGACCTCGTGGCAAAGTACAACACC
s-DAPK-1        AGCGAG-----------------------------TGAGTTTGTGGAAAGATAAGAGAC-
                  *  *                             ***  * **** **  **  * **

DAPK-1          AGTAACGGGGCTCCCAAGGATTTCCTCCCCAGCCCCCTCCACGCCCTGCTGCGGGAATGG
s-DAPK-1        ----------CTTCCAAGAGTTTCCT----------------------------------
                          ** *****  ******

DAPK-1          ACCACCTACCCTGAGAGCACAGTGGGCACCCTCATGTCCAAACTGAGGGAGCTGGGTCGC
s-DAPK-1        ---------------------------------------AAACCAGGACAGTTTTGT---
                                                       ****   *  ** *  **

DAPK-1          CGGGATGCCGCAGACTTTTTG-CTGAAGGCATCCTCTGTGTTCAAAATCAACCTGGATGG
s-DAPK-1        -------------GCTTTGTGCCTTGATTCATTCTCTGTATATGATGTCA--CAGAAGAG
                              **** ** **  *  *** ****** *   *  ***  * * *  *
```

```
DAPK-1          CAATGGCCAGGAGGCCTATGCCTCGAGCTGCAACAGCGGCACCTCTTACAATTCCATTAG
s-DAPK-1        CC---------AGGTCTGTTCCCCAGGTTGATGCTAAAG-----TTTGTGTTTCTCTTAC
                *          *** ** * ** *  * **   *    *      **    ***  ***

DAPK-1          CTCTGTTGTATCCCGGTGAGGGCAGCCTCTGGCTTGGGCAGGGTCTGTTTGGACTGCAGA
s-DAPK-1        GTCC------CCTCAGTGAGGGAGGACGCTGG--------------------------GG
                 **        * * *******  * * ****                          *

DAPK-1          AGCAAGGGGGTGATGTAGCCCATCCTTCCCTTTGGAGATGCTGAGGGTGTTTCTTCCTGC
s-DAPK-1        AGCATGG-------GTAGC-------------------TGCGGAGAG-------------
                **** **       *****                   *** *** *

DAPK-1          ACCCACAGCCAGGGGGATGCCACTCCTCCCTCCGGCTTGACCTGTTTCTCTGCCGCTACC
s-DAPK-1        ------------------------------------------------TTGGCTGAGATT
                                                                *  ** *  *

DAPK-1          TCCCTCCCCGTCTCA--TTCCGTTGTCTGTGGATGGTCATTGCAGTTTAAGAGCAGAACA
s-DAPK-1        TATATTCCCTTTTCAACTTCTGTTCTCTG---------AACACAGCTATAGAATTGAAA-
                *   * *** * ***  *** *** ****         *   *** *  ***   ***

DAPK-1          GATCTTTTACTTTGGCCGCTTGAAAAGCTAGTGTACCTCCTCTCAGTGTTTTGGACTCCA
s-DAPK-1        --------------------TGAGGAG---------------------------------
                                    ***  **

DAPK-1          TCTCTCATCCTCCAGTACCTTGCTTCTTACTGATAATTTTGCTGGAATTCCTAACTTTTC
s-DAPK-1        ------------------------------------------------------------

DAPK-1          AATGACATTTTTTTTAACTACTATATTGATTGTCCTTTAAAAAAGAAAAGTGCATATTTA
s-DAPK-1        --------------------------------------AAGAAAGAA-----------TA
                                                      ** ******           **

DAPK-1          TCCAAAATGTGTATTTCTTATACGCTTTTCTTTGTTATACCATTTCCTCAGCTTATCTCT
s-DAPK-1        ACCAAAACAGA----------------------GTTATAA--------------------
                 ******                          ******

DAPK-1          TTTATATTTGTAGGAGAAACTCCCATGTATGGAATCCCACTGTATGATTTATAAACAGAC
s-DAPK-1        TTCATATT------------------------------ATTATATTGCTGATA-------
                ** *****                              * * ***   * ***

DAPK-1          AATATGTGAGTGCCTTTTGCAGAAGAGGGTGTGTTTGAAATCATCGGAGTCAGCCAGGAG
s-DAPK-1        CATATGTCAATA---TTTATAGAGTACTGTGT--------------------GCCAGGAA
                 ****** * *    ***  ***  *  ****                    *******

DAPK-1          CTGTCACCAAGGAAACGCTACCTCTCTGTCCCTTGCTGTATGCTGATCATCGCCAGAGGT
s-DAPK-1        CTG-----GAAGAAA---------------------TATGGTCTGAAGGACGCC-----T
                ***      * ****                     * *   ****    ****     *
```

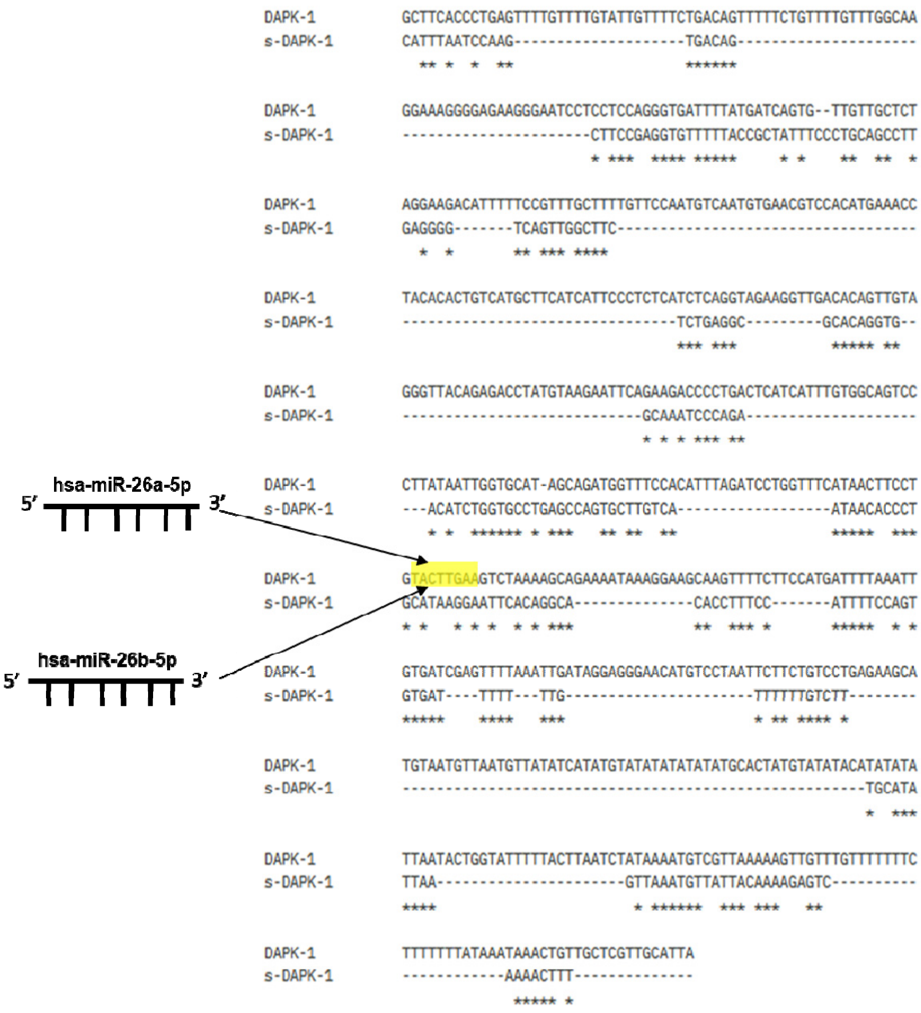

Figure S2. The sequences of miRs together with their target sequence (highlighted in yellow) on s-DAPK-1 and DAPK-1 mRNA. Symbols: asterisk (*) indicates a position where the sequences being aligned have conserved residues, a gap (-) represents a missing or extra region of a sequence that is present in only one sequence.

Supplementary S3

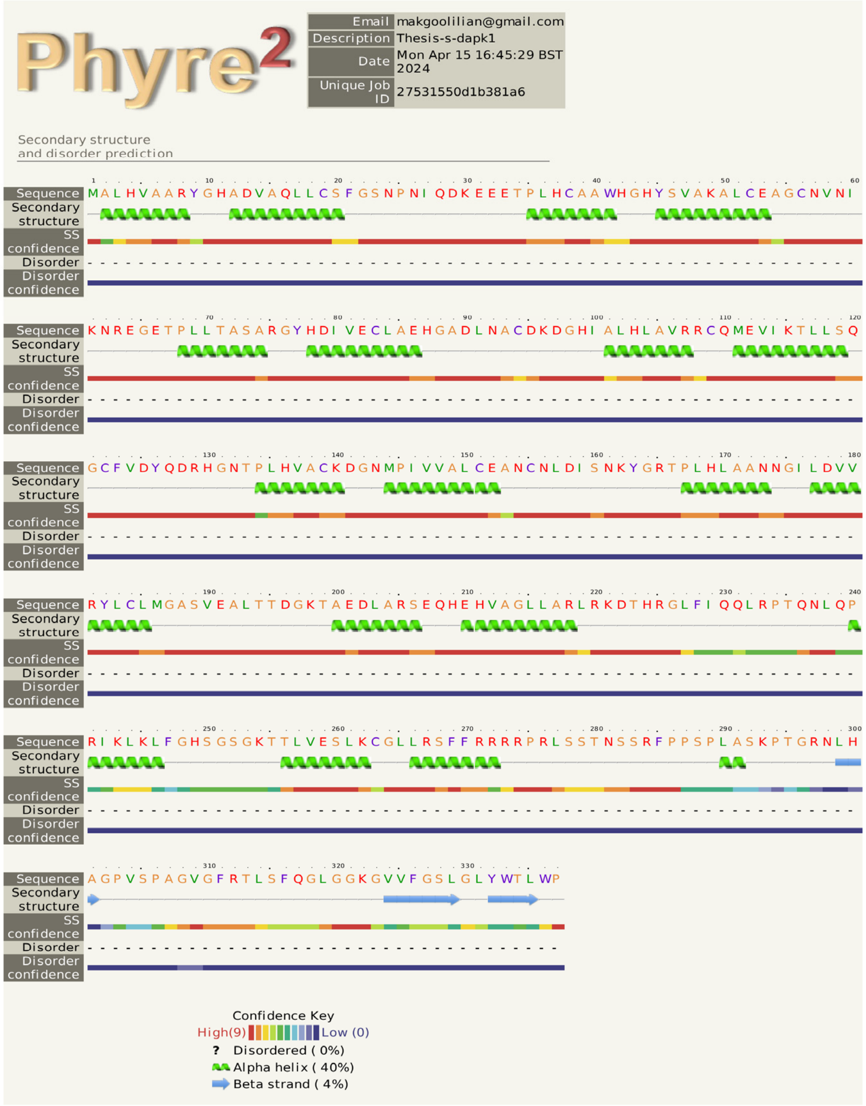

Figure S3. The predicted secondary structure of s-DAPK-1 using the Phyre2 tool.

Supplementary S4

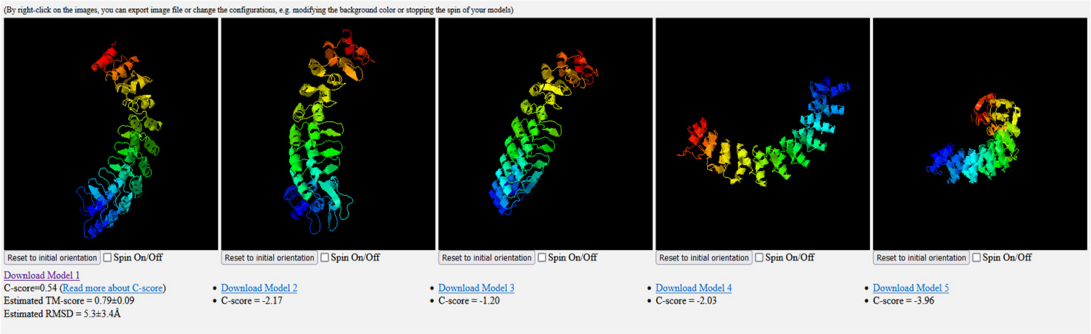

Figure S4. Predicted modeled 3D structures of s-DAPK-1 with varying C-scores. On the predicted protein structures, the red secondary structures are at the c-terminus of s-DAPK-1 while the blue structures are at N-terminus.

Supplementary S5

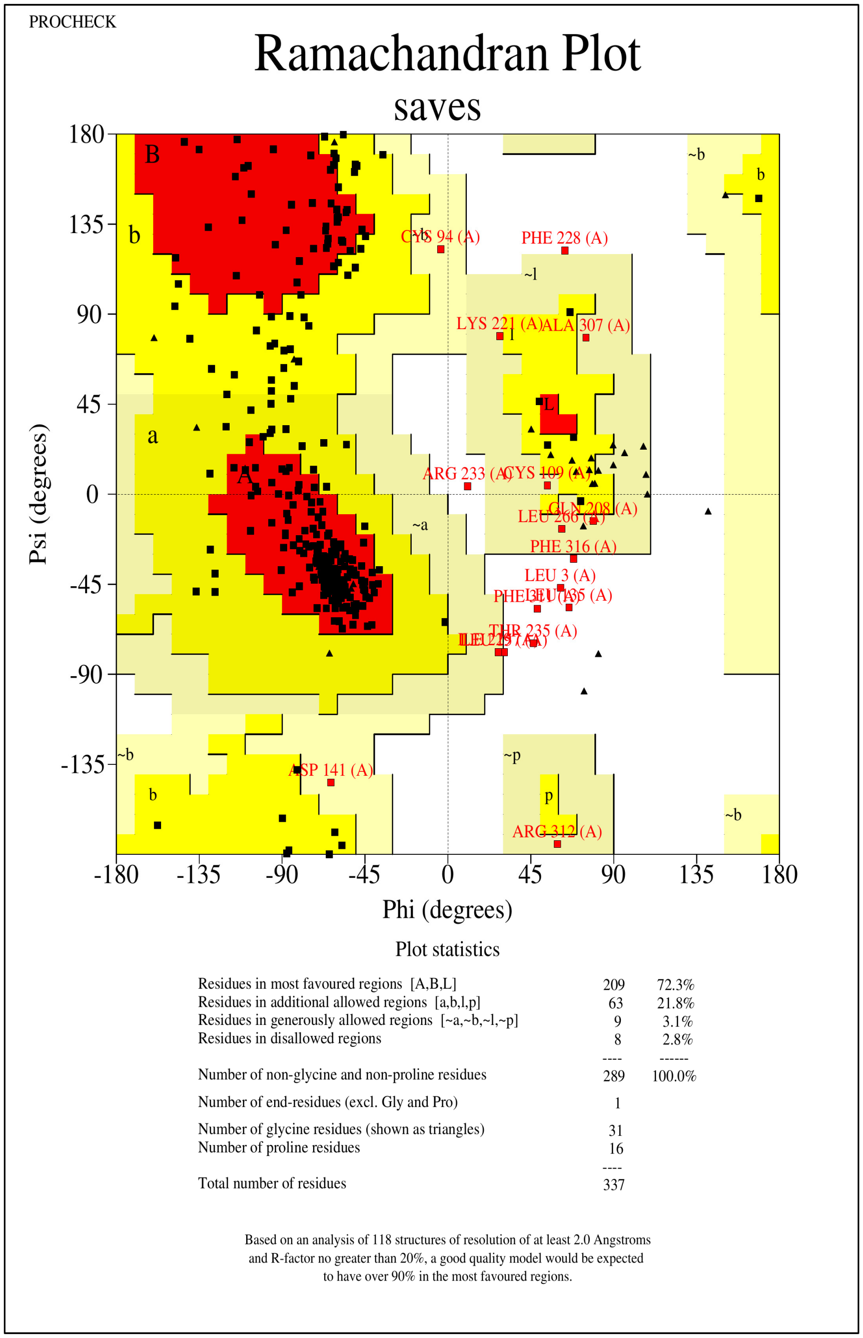

saves_01.ps

Figure S5. Ramachandran plot. Glycines are represented as triangles and any other amino acids are symbolised as square. Colour shades: red indicates amino acids in most favoured regions, yellow indicates amino acids in additional allowed regions, light yellow indicates amino acids in generously allowed regions and lastly white indicates amino acids in disallowed regions.
